# Atypical perioperative management for duodenal obstruction in an infant with heterotaxy syndrome: a case report

**DOI:** 10.1186/s40981-018-0154-5

**Published:** 2018-02-12

**Authors:** Hirofumi Obinata, Shinichi Nishibe, Yoko Ishihara

**Affiliations:** 1 Department of Anesthesiology, Saitama International Medical Center, 1397-1 Yamane, Hidaka, Saitama Japan; 2 Department of Anesthesiology, Tokyo Metropolitan Children’s Medical Center, 2-8-29 Musashidai, Fuchu, Tokyo Japan; 3 Department of Anesthesiology, Self-Defense Forces Central Hospital, 1-2-24 Ikejiri, Setagaya, Tokyo Japan

**Keywords:** Preduodenal portal vein, Heterotaxy, Intestinal malrotation, Total anomalous pulmonary venous connection, Duodenal obstruction

## Abstract

**Background:**

Heterotaxy syndrome (HS) is characterized by a wide variety of cardiac and extra-cardiac malformations, including pulmonary valve stenosis, interruption of the inferior vena cava, total anomalous pulmonary venous connection (TAPVC), asplenia, polysplenia, intestinal malrotation, and preduodenal portal vein (PDPV). We report the case of a heterotaxic infant with an infracardiac TAPVC and preduodenal portal vein who experienced repetitive hemodynamic instability during urgent laparotomy for duodenal obstruction.

**Case presentation:**

A 3-day-old boy with HS was planned to undergo urgent laparotomy for duodenal atresia. Echocardiogram showed an interrupted inferior vena cava, single right ventricle, pulmonary valve stenosis, and infracardiac TAPVC. On exploratory laparotomy, intestinal malrotation characterized by Ladd’s band was found. During further exploration, repetitive severe hypotension and hypoxia occurred. Thorough examination revealed a greatly dilated PDPV crossing over and compressing the proximal duodenum externally. Finally, we considered the possibility that surgical manipulation directly compressed the dilated PDPV into which the TAPVC had pulmonary venous drainage, leading to repetitive pulmonary venous obstruction (PVO). Computed tomography, which was examined after laparotomy, indicated that the vertical vein from pulmonary venous confluence drained into the portal vein.

**Conclusion:**

PDPV is a rare anomaly associated with HS. In case of intestinal malrotation and duodenal obstruction in HS with infracardiac TAPVC, both the presence of PDPV and the possibility of pulmonary venous drainage into the PDPV should be considered by pediatric surgeons and anesthesiologists performing laparotomy to avoid catastrophic PVO.

## Background

Heterotaxy syndrome (HS) is characterized by a wide variety of cardiovascular malformations, including pulmonary valve stenosis, interruption of the inferior vena cava, and total anomalous pulmonary venous connection (TAPVC) and is also associated with various extra-cardiac congenital malformations, including asplenia, polysplenia, midline liver, and intestinal malrotation [1]. In the case of infracardiac TAPVC, the confluence of pulmonary veins drains into a vertical vein which enters the systemic venous bed below the diaphragm. The most common site of drainage is the portal vein [2].

Preduodenal portal vein (PDPV) is a rare anomaly in which the portal vein passes anterior to the duodenum rather than posteriorly, mostly seen in heterotaxy and situs inversus [3]. PDPV may be associated with or cause duodenal obstruction [3, 4].

We report the case of heterotaxy in an infant with an infracardiac TAPVC who underwent urgent laparotomy for duodenal obstruction. Laparotomy revealed an intestinal malrotation and PDPV extrinsically compressing the duodenum. During the procedure, there was repetitive hemodynamic instability due to pulmonary venous obstruction (PVO) caused by direct surgical manipulation of the dilated PDPV into which the TAPVC had pulmonary venous drainage.

## Case presentation

This case report was approved by our institutional review board (Saitama International Medical Center 16-074), and the requirement of permission for publication from the parents of the patient was waived.

A male infant with prenatal diagnosis of HS (asplenia) associated with infracardiac TAPVC, interrupted inferior vena cava, single right ventricle and pulmonary valve stenosis, and duodenal atresia was born at 37 weeks of gestation weighing 2980 g. He was referred to our hospital when he was 1 day old. Upon admission to our hospital, his general condition was stable. He did not need any inotropic agent or mechanical ventilation. Baseline blood pressure, heart rate, pulse oximeter readings, and respiratory rate were 55–70/40 mmHg, 110–140 beats/min, 80–90% in room air, and 30–50 /min, respectively. The stomach was decompressed through an orogastric tube.

When he was 3 days old, urgent laparotomy for duodenal atresia was planned prior to TAPVC repair, because preoperative PVO appeared to be mild.

General anesthesia was induced with midazolam (0.7 mg), fentanyl (10 μg), and rocuronium (6 mg) with 100% oxygen. After tracheal intubation, general anesthesia was maintained with 1% sevoflurane, 0.3 μg/kg/min of remifentanil, and 10 μg/kg/min of rocuronium. After intubation, inspired oxygen concentration (FiO_2_) was maintained at 21% and PaCO_2_ was targeted at 40–45 mmHg. Dopamine infusion was started at 3 μg/kg/min.

On exploratory laparotomy, intestinal malrotation characterized by Ladd’s band was found. During further exploration for duodenal obstruction, repetitive severe hypotension (e.g., from 70/36 to 40/28 mmHg) and oxygen desaturation (e.g., from 84 to 63%) occurred despite escalation of FiO_2_ (100%) and dopamine infusion (5 μg/kg/min). Temporary suspension of surgery was often required to improve the status. We then informed the surgeon of the possibility of PVO occurrence due to surgical manipulation. Thorough examination revealed a greatly dilated PDPV, which had been misjudged as being the duodenum, crossing over and compressing the proximal duodenum externally. Finally, we considered the possibility that surgical manipulation directly compressed the dilated PDPV into which the TAPVC had pulmonary venous drainage, leading to repetitive PVO. Ladd procedure with gastroduodenostomy, bypassing PDPV, was done on the basis of the diagnosis of intestinal malrotation and duodenal stenosis due to extrinsic compression by PDPV.

**Fig. 1:**
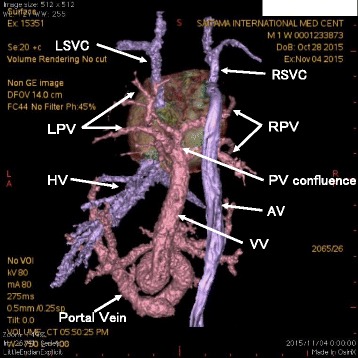
Computed tomography angiography shows that the vertical vein from pulmonary venous confluence goes through the diaphragm and drains into the portal vein, winding its way like a spiral. *LSVC* left superior vena cava, *RSVC* right superior vena cava, *LPV* left pulmonary vein, *RPV* right pulmonary vein, *HV* hepatic vein, *AV* azygous vein, *VV* vertical vein

After completion of surgery, the patient was admitted to intensive care unit with 0.3 μg/kg/min of milrinone and 3 μg/kg/min of dopamine. No postoperative hemodynamic instability was observed. One month later, TAPVC repair and modified Blalock–Taussig shunt was performed. Computed tomography prior to cardiac surgery indicated that the vertical vein drained into the portal vein (Fig. 1).

### Discussion

In this report, we present a rare case of heterotaxy in an infant with infracardiac TAPVC and duodenal obstruction due to external compression by a dilated PDPV, into which infracardiac TAPVC had pulmonary venous drainage. Repetitive hemodynamic instability occurred due to severe PVO caused by direct surgical compression of the PDPV, i.e., the drainage site of the vertical vein from the confluence of pulmonary veins. Although PDPV is seldom diagnosed preoperatively, its potential presence should be considered by pediatric surgeons and anesthesiologists performing laparotomy in patients with intestinal obstruction, especially in those affected by malrotation or abnormal position of viscera (heterotaxy) [5].

Hemodynamic goals before surgical correction of TAPVC include maintenance of cardiac output and avoidance of worsening pulmonary edema. Increased pulmonary blood flow can be detrimental in case of obstructed pulmonary venous flow, by causing worsening pulmonary edema in the face of a fixed obstruction [6]. Therefore, we attempted to avoid FiO_2_ escalation and hypocapnia occurrence during surgery. However, we were obliged to escalate FiO_2_ (100%) and dopamine infusion (5 μg/kg/min) with correction of metabolic acidosis to manage the severe obstruction caused by direct compression of the PDPV. In case of medical treatment-resistant hypoxia, hypotension, and pulmonary hypertension due to severe PVO, especially that caused by mechanical obstruction leading to irreversible vascular damage, emergency establishment of extracorporeal membrane oxygenation must be considered.

Intestinal obstruction is the most common surgical emergency in the neonatal period.

High mortality (18%) was reported after the emergency Ladd procedure for intestinal malrotation with heterotaxy [7]. On the other hand, neonates with infracardiac TAPVC presenting severe hypoxia and acidosis should be immediately taken to the operating room [1]. In our case, laparotomy for duodenal obstruction was performed prior to mildly obstructed TAPVC repair because the fluid and electrolyte imbalance caused by intestinal obstruction can aggravate the fragile hemodynamic state of the patient with infracardiac TAPVC. Juvekar et al. [8] reported combined surgery for obstructed infracardiac TAPVC and malrotation volvulus. According to their report, the surgery was successfully performed at the expense of intensive postoperative care. A staged procedure should be considered if the intestinal malrotation is not associated with volvulus or if PVO is sufficiently mild. Therefore, the timing of surgery in patients with a combination of TAPVC and intestinal obstruction should be discussed among physicians including a pediatric surgeon, a cardiac surgeon, a cardiologist, and an anesthesiologist.

## Conclusions

PDPV is a rare anomaly associated with HS. In case of intestinal malrotation and duodenal obstruction in HS with infracardiac TAPVC, both the presence of PDPV and the possibility of pulmonary venous drainage into the PDPV should be considered by pediatric surgeons and anesthesiologists performing laparotomy to avoid catastrophic PVO.

## Abbreviations

HS: Heterotaxy syndrome
PDPV: Preduodenal portal vein
PVO: Pulmonary venous obstruction
TAPVC: Total anomalous pulmonary venous connection
